# fcScan: a versatile tool to cluster combinations of sites using genomic coordinates

**DOI:** 10.1186/s12859-020-3536-4

**Published:** 2020-05-19

**Authors:** Abdullah El-Kurdi, Ghiwa Ali Khalil, Georges Khazen, Pierre Khoueiry

**Affiliations:** 1 Department of Biochemistry and Molecular Genetics, Faculty of Medicine, American University of Beirut, Beirut, Lebanon; 2 Pillar Genomics Institute, Faculty of Medicine, American University of Beirut, Beirut, Lebanon; 3 Department of Computer Science and Mathematics, Lebanese American University, Byblos, Lebanon

**Keywords:** Genome scan, Genomic clusters, Cis-regulatory modules, Next generation sequencing, Bioconductor, Variants data, Transcription factor binding sites, GRanges, Vcf, Bed

## Abstract

**Background:**

Finding combinations of homotypic or heterotypic genomic sites obeying a specific grammar in DNA sequences is a frequent task in bioinformatics. A typical case corresponds to the identification of cis-regulatory modules characterized by a combination of transcription factor binding sites in a defined window size. Although previous studies identified clusters of genomic sites in species with varying genome sizes, the availability of a dedicated and versatile tool to search for such clusters is lacking.

**Results:**

We present fcScan, an R/Bioconductor package to search for clusters of genomic sites based on user defined criteria including cluster size, inter-cluster distances and sites order and orientation allowing users to adapt their search criteria to specific biological questions. It supports GRanges, data frame and VCF/BED files as input and returns data in GRanges format. By performing clustering on vectorized data, fcScan is adapted to search for genomic clusters in millions of sites as input in short time and is thus ideal to scan data generated by high throughput methods including next generation sequencing.

**Conclusions:**

fcScan is ideal for detecting cis-regulatory modules of transcription factor binding sites with a specific grammar as well as genomic loci enriched for mutations. The flexibility in input parameters allows users to perform searches targeting specific research questions. It is released under Artistic-2.0 License. The source code is freely available through Bioconductor (https://bioconductor.org/packages/fcScan) and GitHub (https://github.com/pkhoueiry/fcScan).

## Background

Biological information encoded in the genome is organized in modular structures or clusters. For instance, enhancers are structured in cis-regulatory modules (CRMs) characterized by the presence of a combination of homotypic or heterotypic transcription factor binding sites (TFBS) and having, in some cases, a well-defined order and orientation [1–3]. Similarly, several diseases, mainly cancer, are characterized by the presence of clusters of DNA mutations associated with disease appearance and progression [4–6].

Although many bioinformatic approaches rely on the identification of combinations of genomic features, the availability of a tool to search for such combinations, with increased flexibility in searching criteria, is lacking.

For instance, bedtools [7] is an extremely efficient genome arithmetic tool with several commands, including the “merge” and “cluster” tools, for manipulating genomic sites using different input formats. It allows the clustering/merging of sites within a certain range but without controlling the order and orientation of sites within identified clusters or the ability to exclude clusters having a specific site. Several subsequent filtering and scripting steps will be needed to obtain the final set of clusters with the desired combination and window size. Similar to bedtools are the Bioconductor/R packages that enable series of operations for joining and merging sites from GenomicRanges, plyranges and valr [8–10].

A more dedicated tool for genomic clusters identification with flexibility in defining sites grammar is SECOMOD [1]. Although efficient, SECOMOD is tailored to search for CRMs in the compact genome of the sea squirt *Ciona intestinalis* (renamed to *C. robusta*) by using exclusively DNA sequence motifs defined in IUPAC code. It depends on several prerequisites to run including a preformatted reference genome, the availability of a whole genome alignment in xmfa format with conservation scores to detect evolutionary conserved clusters and a motif with a well-defined IUPAC code to scan the genome for putative hits. The multitude of prerequisites made SECOMOD hard to implement for other genomes, mainly for large mammalian genomes.

Here we present fcScan, an R/Bioconductor package to scan DNA sequences for clusters of genomic features based on user defined criteria. It takes as input genomic coordinates representing any type of genomic feature including TFBS (identified using IUPAC motifs or Position Weight Matrices (PWM)), in-vivo ChIP-seq data, Transcription Start Sites (TSSs), Alu repeats and Single Nucleotide Polymorphisms (SNPs) in several formats and returns a GRanges object thus allowing integration with other R/Bioconductor pipelines. Our tool is sequence and genome independent and its application on simulated and real biological data highlight its computing performance and ability to detect real biological modules.

## Implementation

**Fig. 1:**
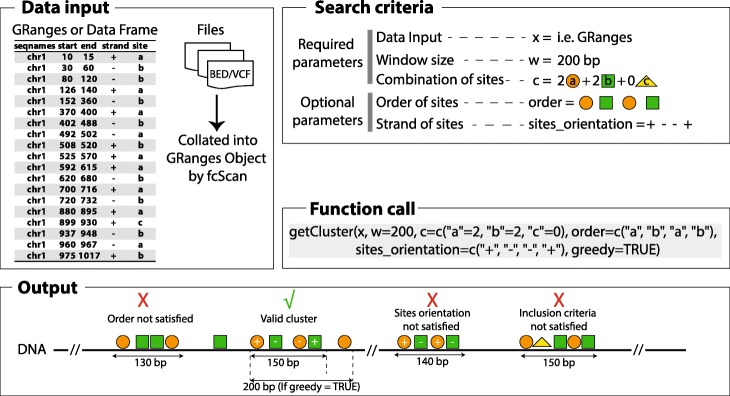
fcScan search strategy and performance. Left: Example of data input that can be one of GRanges, data frame or BED/VCF files. Right: schematic representation of a search example for clusters containing a combination of 2 heterotypic genomic features (orange circles and green squares) and excluding a third genomic feature (yellow triangle) in a window size of 200 bp. Sites within identified clusters must obey the order and orientation defined. Bottom: Function call on the data represented in the upper left corner with its corresponding output. Only one cluster, marked by the “correct” sign, is called based on the criteria above. All remaining clusters are eliminated for the reason described above each case

Features Cluster Scan (fcScan) is implemented in the R programming language and follows the guidelines defined by the Bioconductor community. The main function is called “getCluster” with three required arguments corresponding to input data, window size and clustering conditions (Fig. 1).

“getCluster” accepts input in GRanges or data frame R objects as well as BED or VCF files. When a GRanges or a data frame object is supplied, a “site” field containing the category name of the genomic feature, and based on which the clustering will be performed, should be defined. When the input is a vector of file paths in BED or VCF formats, “getCluster” imports and collates the files into one GRanges object (Fig. 1).

The maximum size desired for a cluster is defined by the window size argument “w”. For example, as cis-regulatory modules are defined by a combination of TFBS in a typical size range of 200 to 500 bp, the user would define “w” as 500 bp to search for such modules.

The third required argument defines clustering conditions using a named numeric vector “c” indicating the desired number of each site in the identified clusters (Fig. 1). For instance, searching for clusters containing 2 sites “a” and 2 sites “b” and 0 site “c” would be indicated by “c = c(“a” = 2, “b” = 2, “c” = 0)”. This means that users can exclude clusters containing a specific site (i.e. site “c” in this example) by specifying “0” as the desired number.

In the case of a GRanges or a data frame object as input, the “c” vector should be named with names among the site names defined in the “site” column of the data frame.

Alternatively, when the input is a vector of BED or VCF files, naming the conditions vector can be ignored and fcScan will attribute numbers from 1 onward as names for sites in each file in the order of input files. For instance, in the following function call “*getCluster(x = c(“file 1.bed”, “file 2.bed”), c = c (2, 2), w = 200)”*, fcScan will attribute “1” as name for sites from file 1.bed and “2” for sites from file 2.bed.

### Optional input arguments

In order to include nearby putative functional sites in detected clusters, we defined the “greedy” logical argument. When set to “TRUE”, “getCluster” will include as many sites as possible, referred as “subclusters”, until the specified window size is reached (Fig. 1). Even when the condition on the combination of sites has been met, if the cluster size is still smaller than the requested size, getCluster will continue adding new sites to the cluster.

To control gaps and overlaps between adjacent clusters, we specified the “overlap” argument that defines the distance or gap allowed between identified adjacent clusters. When set to a negative value, for example − 20, the user allows overlap between adjacent clusters of maximum 20 bp. Conversely, when set to a positive value, for example 20, the user requires for adjacent clusters to be separated by a minimum distance of 20 bp.

The “order” and “sites_orientation” arguments define the order and strand orientation of the sites within a cluster. The argument “sites_orientation” can only be defined if the “order” argument is defined. When greedy is set to “TRUE”, the presence of a “subcluster” satisfying the order and orientation is enough to pass the test (Fig. 1).

Additionally, fcScan allows identifying clusters in a subset of input data by restricting the search to specific seqname(s) or strand assuming strand information is available in the input data.

## Results and discussion

**Fig. 2:**
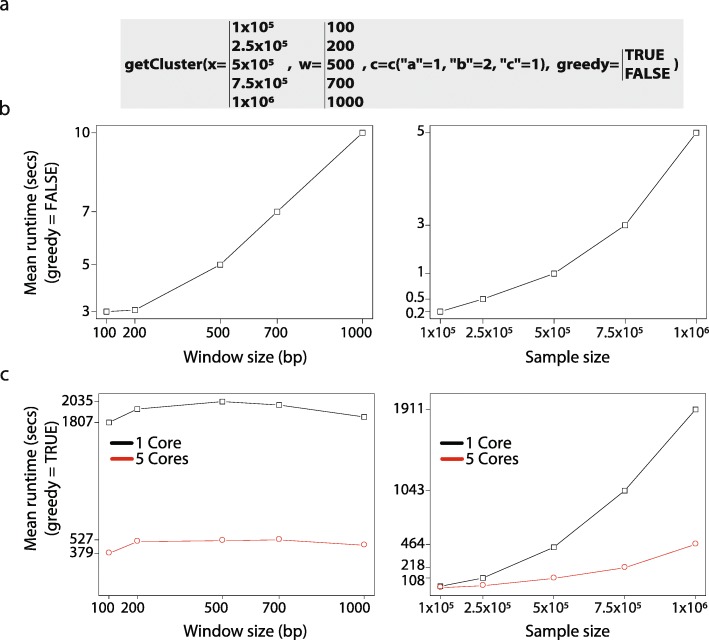
fcScan is optimized to scan and cluster large input data. **a** Call used for benchmarking “getCluster” function in fcScan on a randomly generated datasets of up to 1,000,000 features and 3 heterotypic genomic features (**a**, **b**, and **c**) over 5 chromosomes using arguments “c = c(“a” = 1, “b” = 2, “c” = 1)” with “greedy = FALSE” for panel “b” and “greedy = TRUE” for panel “c” with either 1 or 5 computing cores (1 computing core per chromosome). For input “x”, window size “w” and “greedy” options, all different used values are shown. **b,c** Left panels corresponds to a fixed number of input size of 1,000,000 features as a function of window size. Right panels correspond to a fixed window size of 500 bp as a function of input size. Y-axis represents rounded mean runtimes in seconds over 10 runs

Several Next Generation Sequencing (NGS) assays represent their data in genomic coordinates including ChIP-seq on transcription factor for the identification of in-vivo binding events and whole exome or whole genome sequencing for variants discovery. Since fcScan is optimized to search for combination of sites with a specific grammar in large datasets, it is ideal to deal with NGS related data. To test the runtime of our algorithm, we sampled a random dataset of 1,000,000 entries corresponding to 3 heterotypic binding sites (a, b and c) with a size range between 7 bp and 10 bp over 5 chromosomes. We then used the package “microbenchmark” from CRAN to benchmark calls of getCluster (the main function in fcScan) and plotted the mean runtime as a function of window size, with a fixed sample size of 1,000,000, or as a function of sample size with a fixed window size of 500 bp (Fig. 2a). When greedy is set to FALSE (Fig. 2b), runtime increases almost linearly with increasing window size (left) or sample size (right) but remains, in both cases, relatively short. For instance, it takes 2.7 s to search 1,000,000 sites with a window size of 100 bp (Fig. 2b, left panel). When the greedy option is set to TRUE, runtime was not affected by the window size (Fig. 2c, left panel) but was constantly larger compared to the non-greedy call above (1802 s with greedy = TRUE as opposed to 2.7 s with greedy = FALSE). However, runtime was reduced by approximately 5 folds when the function call was parallelized over 5 cores (as our dataset contains features over 5 chromosomes) (Fig. 2c, left panel) highlighting the importance of parallelizing the search specifically when greedy is set to TRUE.

In contrast to window size increase, runtime was affected by sample size increase when the greedy option was set to TRUE (Fig. 2c, right panel). Again, parallelizing the function call over several cores showed a dramatic decrease in runtime.

Next, we evaluated the performance of fcScan on actual biological data. We chose to detect the clusters at the boundaries of topologically associated domains (TADs) characterized by the presence of the CCCTC-Binding factor (CTCF), in forward or reverse orientation, that colocalizes with the Cohesin complex. As a proof of concept, we aimed at identifying CTCF/SMC3 clusters flanking the very well-established TAD containing the Sonic Hedgehog gene (shh) and its conserved ZRS enhancer located on chromosome 7 [11, 12].

For this, we run fcScan using as input CTCF (ENCFF396BZQ) and SMC3 (part of the cohesion complex; ENCFF175UEE) ChIP-seq peaks on K562 from ENCODE [13] as well as CTCF ChIP-seq peaks identified in GM12878 (ENCFF960ZGP) to ensure detection of more stable CTCF/SMC3 clusters. We also added as input SNPs data from COSMIC on chromosome 7 (1,075,437 SNPs) [14] since mutations affecting CTCF binding are known to disrupt TADs, as well as input for CTCF binding sites identified by FIMO [15] at a threshold of 10e^− 5^ using CTCF PWM from Jaspar (MA0139.1, 58,467 sites) [16] to detect the orientation of CTCF binding in the identified clusters.

**Fig. 3:**
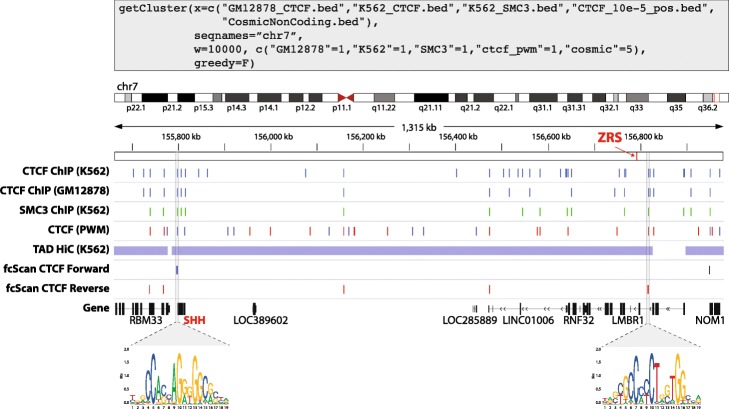
fcScan identified CTCF clusters delimiting the shh/ZRS TAD in Forward and Reverse nomenclatures. Genome browser view showing tracks of CTCF ChIP-seq on K562 and GM12878, SMC3 ChIP-seq on K562, CTCF PWM hits in forward (blue) and reverse (red), Hi-C based TADs in K562 and fcScan identified CTCF clusters for forward (blue) and reverse (red) CTCF sites. The last track shows the gene models with shh gene highlighted in red. CTCF PWM for forward and reverse orientations are shown below. COSMIC data track was omitted for clarity. The function call used to identify the clusters is shown on top and was run independently for CTCF PWM based binding sites on positive (the call showed) and negative strands

Using 10 Kb window size and a combination of 1 site from each of the input sites above except for COSMIC where we requested 5 sites, fcScan took 17.1 and 13.5 s to scan 1,083,788 and 1,083,805 sites for the detection of clusters with forward and reverse CTCF binding sites respectively. This resulted in a total of 431 clusters with forward CTCF orientation (F) and 366 clusters with reverse CTCF orientation (R), exclusive to chromosome 7. We then downloaded the list of TADs identified by Hi-C in K562 from TADKB (3743 TADs) [17] and identified TADs having a CTCF/SMC3 cluster identified by fcScan in a range of 15Kb at both ends of the TAD. Notably, fcScan detected two CTCF/SMC3 clusters flanking the 380Kb shh/ZRS TAD in the FR nomenclature (Fig. 3).

As a second application, we used fcScan to detect known CRMs characterized by a defined combination of transcription factor binding sites. In this case, we chose to identify neural CRMs in the ascidian *Ciona robusta* (*C. intestinalis* previously) characterized by the presence of 2 GATA and 2 ETS binding sites as previously performed using SECOMOD [1]. This second application allows us to compare fcScan performance and output with SECOMOD.

We run fcScan and SECOMOD on a total of 1,048,295 binding sites coordinates for GATA (with GATA as motif) and ETS (with MGGAAR as motif) in a window of 130 bp (With greedy = T for fcScan). The search identified 12,815 and 12,593 non-overlapping clusters by SECOMOD and fcScan, respectively. Four hundred ninety-seven (497) clusters were missed by fcScan. After inspection, all missed clusters have a size above the window size limit allowed by fcScan (Min size = 132 bp, Median size = 134 bp). This is due to the different approaches used to calculate clusters size in each tool with SECOMOD calculating the size from the end of the first site to the start of the last site while fcScan considers the start of the first site to the end of the last site. Conversely, 77 clusters were missed by SECOMOD with a Median size of 130 bp (Max 130 bp).

Most importantly, “fcScan” identified all 40 conserved CRMs reported previously among them the 7 CRMs that show activity in-vivo [1].

Both fcScan case studies above using real biological data highlight its ability to detect biologically meaningful clusters.

Last, “getCluster” function returns a GRanges object containing the identified clusters allowing a fast and easy integration of fcScan with Bioconductor workflows and packages that uses GRanges as input. Moreover, clusters that fail to match the search criteria (i.e. clusters with the correct combination of sites but wrong order) can be returned with a status indicating the cause of exclusion (i.e. orderFail) by using the “verbose = TRUE” argument.

## Conclusions

To our knowledge, fcScan is the only package in R/Bioconductor to search for clusters of sites based on a list of user defined criteria. It is suitable for detecting cis-regulatory modules characterized by clusters of transcription factor binding sites as well as genomic loci enriched for mutations or a combination of both.

More broadly, fcScan can be applied to detect clusters of sites generated using NGS assays including ChIP-seq, exome and whole genome sequencing as well as for sites in any coordinate system (cartesian coordinate systems) that supports position of features. The accompanying Bioconductor vignette contains additional details and is updated regularly. Future releases will extend the list of options giving the user additional options to identify clusters of interests including, for instance, the ability to define the minimum or maximum distance allowed between sites within a cluster.

## Availability and requirements

**Project name**: fcScan.

**Project home page**: https://bioconductor.org/packages/fcScan and (https://github.com/pkhoueiry/fcScan).

**Operating system**(s): Platform independent.

**Programming language**: R.

**Other requirements**: Bioconductor.

**License**: Artistic-2.0.

**Any restrictions to use by non-academics**: Artistic-2.0.

## Data Availability

The source code for this software is freely available through Bioconductor (https://bioconductor.org/packages/fcScan) and GitHub (https://github.com/pkhoueiry/fcScan). The data analyzed during the current study are available at ENCODE for CTCF and SMC3 ChIP-seq peaks https://www.encodeproject.org/, COSMIC for SNPs (https://cancer.sanger.ac.uk/cosmic), TADKB for TADs Hi-C based peaks on K562 (HiC_K562_DI_10kb; http://dna.cs.miami.edu/TADKB/), JASPAR for CTCF PWM (https://cancer.sanger.ac.uk/cosmic). The SECOMOD code and related datasets (i.e. conservation scores, preformatted Ciona genome, whole genome alignment) are available upon request from the authors [1].

## Abbreviations

NGS: Next Generation Sequencing
TFBS: Transcription factor binding sites
CRM: Cis Regulatory Module
TSS: Transcription Start Site
PWM: Position Weight Matrix
TADKB: Topologically Associating Domain Knowledge Base
COSMIC: Catalogue of Somatic Mutations in Cancer
ENCODE: Encyclopedia of DNA Elements
CTCF: CCCTC-Binding factor
ZRS: Zone of polarizing activity regulatory sequence
SECOMOD: Search for Evolutionary Conserved MODules
